# A drop‐in centre for treating mental health problems in children with chronic illness: Outcomes for parents and their relationship with child outcomes

**DOI:** 10.1002/jcv2.12046

**Published:** 2021-10-25

**Authors:** Sophie D. Bennett, Eleanor Kerry, Kate Fifield, Brian C. F. Ching, Matteo Catanzano, Holan Liang, Isobel Heyman, Anna E. Coughtrey, Charlotte Sanderson, Natalia Rojas, Roz Shafran

**Affiliations:** ^1^ UCL Great Ormond Street Institute of Child Health London UK; ^2^ Great Ormond Street Hospital for Children NHS Foundation Trust London UK

**Keywords:** chronic illness, drop‐in, mental health, parents

## Abstract

**Background:**

Children with chronic health conditions and their parents are at greater risk of developing emotional and behavioural problems compared to their physically healthy peers. The psychological impact on parents is crucial to understand given the relationship between parental mental health and child emotional and behavioural difficulties. This study was part of a broader research project examining the acceptability, feasibility and impact of a ‘Mental Health and Psychological Wellbeing Drop‐in Centre’ in a paediatric hospital providing access to support and intervention for children and their families. This paper aimed to investigate the impact of the centre on parents (*n* = 148).

**Methods:**

Parental anxiety and depression were assessed using the GAD‐7 and PHQ‐9 at baseline and 6‐month post‐baseline. Child mental health was assessed using the parent‐report Strengths and Difficulties Questionnaire (SDQ). If parents had significant mental health needs, a brief intervention/signposting to relevant services was provided.

**Results:**

At baseline, 48% of parents scored above clinical threshold for anxiety and 41% for depression, and parent reported child SDQ scores were correlated with parental anxiety and parental low mood. Self‐reported parental anxiety and low mood decreased at 6‐months post‐baseline (parental anxiety: mean decrease = 2.29 [1.22–3.36], *d* *=* 0.38; parental low mood: mean decrease = 1.81 [0.64–3.00], *d* *=* 0.28). There were no significant correlations between change in parent reported child wellbeing and changes in parental low mood and anxiety between baseline and 6‐month post‐baseline.

**Conclusions:**

Assessing and providing a brief treatment to address the mental health needs of parents of children with comorbidity may bring important benefits. It is recommended that children's mental health services consider assessment of parental mental health as part of routine care.

**Key points**

- Children with chronic physical health conditions are at greater risk of developing emotional and behavioural problems compared to their physically healthy peers.
- Parents of children with physical health difficulties also show elevated symptoms of depression and anxiety.
- The findings suggest that having a child with comorbid physical and mental difficulties places a toll on parental mental health.
- Assessing and addressing the mental health of parents of children with comorbidity as part of routine treatment for the child's mental health may bring important benefits to parents and children.

## INTRODUCTION

Children with chronic physical health conditions are at greater risk of developing emotional and behavioural problems compared to their physically healthy peers (Hysing et al., 2007; Pinquart & Shen, 2011a, 2011b, 2011c). This heightened risk is likely the result of a combination of psychological, social and biological factors including shared underlying pathology, increased uncertainty, peer relations, changes in parenting style and the side‐effects of medication (Holmbeck et al., 2002; Miller et al., 2008; Sandstrom & Schanberg, 2004). Children with psychological needs may also struggle more with treatment compliance and self‐management of their health which both in turn may lead to a reduced quality of life in the long‐term (Hood et al., 2006; Lima et al., 2013). It is increasingly recognised that having physical and psychological needs creates greater demand for services and therefore has a financial impact on the healthcare system (Naylor et al., 2012).

Parents of children with physical health difficulties also show elevated symptoms of depression and anxiety (Pinquart, 2019a, 2019b). Studies suggest that these parents are under increased strain from balancing work and family commitments whilst also needing to manage their own anxieties about their child's future (Barlow & Ellard, 2006; Cousino & Hazen, 2013). The psychological impact on parents is particularly crucial to understand given the bi‐directional relationship between parental mental health and child emotional and behavioural difficulties (Bagner et al., 2013; Stone et al., 2016). Genetically informed studies have highlighted the importance of environmental factors and parenting in the intergenerational transmission of anxiety (Eley et al., 2015), depression (McAdams et al., 2015) and conduct problems (D'Onofrio et al., 2007). Parents with mental health difficulties may find it more difficult to manage their children's emotions and behaviours which may have negative consequences for their children's physical and psychological wellbeing (Pierce et al., 2020; Reupert et al., 2013).

Psychological interventions have been successful in targeting distress among parents of children with a range of medical conditions such as cancer, traumatic brain injury, and asthma (Law et al., 2019). Research has also shown that treating parental mental health impacts positively on both parent mental health and child emotional and behavioural problems (Neece, 2014). Some research now also suggests that treating child mental health problems may lead to more positive outcomes for the parents' mental health (Creswell, Violato, et al., 2020; Jarosz & Bayer, 2017; Wilkinson et al., 2013). These findings suggest that it may not be necessary to treat child and parent separately to affect significant positive change for both.

Despite evidence demonstrating adverse outcomes for both parents and children with mental health difficulties in the context of chronic conditions, many are still not accessing evidence‐based treatments for their mental health needs (Acri & Hoagwood, 2015; Bennett et al., 2015, 2018). This unmet need may be in part due to pressures within the family, such as time‐constraints and the cost of travel (Cousino & Hazen, 2013; Sav et al., 2015). However, structural barriers also exist within the healthcare system leading to long waits for treatment, rejected referrals when not meeting service criteria, and a ‘postcode lottery of provision’ (Crenna‐Jennings & Hutchinson, 2020).

A proposed method for overcoming the barriers to access for children with physical and mental health needs, is a ‘mental health drop‐in centre’ located within a paediatric hospital setting. This format of delivery may bring benefits both in terms of the immediacy of the support, but also in terms of the quality of treatment. For instance, being situated in a multi‐disciplinary hospital setting allows for joint‐working and information‐sharing between professionals from different specialties, improvement of referral pathways and increased speed of access to evidence‐based treatments (Tyler et al., 2017). Furthermore, the informality and flexibility of the drop‐in approach may appeal to children and families who might otherwise be discouraged by complicated referral pathways and long waiting times (Anderson et al., 2017). A drop‐in may also allow for earlier detection and intervention, relieving the pressure on more specialist services which would in turn lead to economic benefits for the national health service (Arango et al., 2018; Knapp et al., 2011). The advantages of mental health drop‐in centres have been reported in the general population (Barwick et al., 2013; Nath et al., 2016; Settipani et al., 2019), however, studies have yet to explore the use of a mental health drop‐in centre in a paediatric hospital setting.

This study was part of a broader research project examining the acceptability, feasibility and impact of a ‘Mental Health and Psychological Wellbeing Drop‐in centre’ in a tertiary paediatric hospital setting. A separate study reports on the psychological outcomes for the children accessing the centre (Catanzano et al., 2020). Overall, the drop‐in centre was found to improve mental health and quality of life in children and young people accessing the drop‐in centre. The present study aimed to investigate the impact of the drop‐in on parents; specifically (i) the prevalence of mental health difficulties in parents attending the drop‐in, (ii) the association between parent mental health and child mental health and quality of life at baseline and change over time, and (iii) whether attendance at the drop‐in was associated with change in parental mental health. Whilst existing research has demonstrated the level of mental health needs in parents of children and young people with chronic physical health conditions, there is no research regarding the prevalence in parents who ‘drop‐in’ to such a centre. It is possible that the prevalence may be greater overall as they may either have a child with a mental health need, which itself may be associated with parental mental health difficulties, or because they are accessing the centre for their own mental health needs. Given the anticipated high rates of parental mental health difficulties in this group, it is also important to understand whether such a drop‐in may be associated with improvement in parents' own mental health as well as their child's, and whether this association was the same when parents received a signposting intervention from the drop‐in centre. Such information would provide preliminary evidence for whether such signposting interventions for parents are beneficial. Thirdly, given previous research demonstrating an association between parent and child mental health, we aimed to further understand this relationship at baseline, to determine whether our results replicated those of other samples demonstrating a positive correlation between parent and child mental health. We then wanted to extend this research through examining the correlation between change in child mental health and change in parent mental health. These correlations may help us to consider whether change in child mental health may be a causal or moderating factor in change in parental mental health in order to inform future controlled studies of interventions for either child or parent mental health in populations of children and young people with chronic physical illness.

## METHODS

### Design

This study was part of an uncontrolled trial of a ‘psychological wellbeing drop‐in centre’ for young people, their siblings and carers attending a national paediatric hospital (Catanzano et al., 2020).

### Participants

For the drop‐in centre study, we included patients attending the paediatric hospital for a physical health condition within the last 6‐month and their parents/carers and siblings. The main inclusion criteria was a common mental health need (anxiety, depression and/or behavioural difficulties) that was interfering with current functioning. Parents/carers could receive support from the booth for their own mental health needs as well as their child's. Families must not have been under the care of paediatric psychology services within the hospital at the time and needed to process a sufficient grasp of English to facilitate engagement with the assessment and treatment processes.

### Procedure

Recruitment took place from March 2018 to December 2019. The drop‐in centre served both as a focus for recruitment and for raising awareness of the project. One volunteer/member of staff was present Monday–Friday (10 am–12 pm and 2–4 pm) with a clinical psychologist and/or psychiatrist on call at all times. Participants were recruited into the study via five routes: (i) the family/patient could approach a staff member at the centre (‘physical drop‐in’), (ii) contact the team by e‐mail/telephone, (iii) a staff member could approach a family/patient in other areas of the hospital with a leaflet about the project (‘active recruitment’), (iv) clinicians within the hospital could signpost or (v) refer patients and/or families to the project. Families then consented and completed baseline measures, either in person or over the telephone. Following this, an initial triage assessment, taking approximately 30‐min, was carried out in accordance with a standardised protocol either over the telephone or face‐to‐face depending on participant preference. All patients were then discussed in a weekly meeting with a consultant child and adolescent psychiatrist and allocated to an intervention based on a clinical decision making algorithm that considered factors including clinical risk, relationship to physical health condition, participant preference, neurodevelopmental factors, family factors and symptom severity. At no point in the study was there a waiting list for the initial triage assessment. Decisions were made at a weekly meeting, therefore, participants waited a maximum of seven days to be allocated to an intervention. All measures were completed at baseline after consent, and at 6‐months from baseline. Outcome measures were collected face‐to‐face or remotely by phone/e‐mail (depending on participant preference) by a researcher that was not involved in the delivery of the intervention. All parents/carers completed measures of their own mental health at baseline and 6‐month follow‐up, whether seeking treatment for their child or themselves.

### Ethics

Written informed consent was taken for all participants included in the study by research assistants. In some instances, participants verbally consented over the phone, which was recorded and the responses were written up by research assistants. All methods of data collection were in the ethically approved protocol. Ethics approval was granted by the London Riverside Research Ethics Committee (REC reference number: 16/LO/1915).

### Intervention

Participants were allocated to interventions according to the decision‐making algorithm. Specifically, they could be allocated to the following:

#### Children (hospital patients and siblings) with mental health needs

(i) Provision of/direction to self‐help materials and/or online resources
(ii) Further assessment in the form of either a neurodevelopmental assessment and/or computerised mental health diagnostic assessment (the Development and Wellbeing Assessment; Goodman et al., 2000)
(iii) Signposting/referral to appropriate internal or external services
(iv) A brief modular psychological intervention defined as up to six sessions (6 h total) of either telephone or face‐to‐face guided self‐help based on the Modular Approach to Therapy for Children with Anxiety, Depression, Trauma, or Conduct Problems (MATCH‐ADTC; Chorpita & Weisz, 2009) provided by newly qualified clinical psychologists, trained psychological wellbeing practitioners (i.e., individuals trained specifically in low‐intensity therapies, usually through a specific program, associated with Improving Access to Psychological Therapies initiative) and/or a junior doctor with specific training in the intervention. The intervention was delivered to either the parents or child depending on the child's primary difficulty, age and intellectual ability. For further details of this intervention, see Catanzano et al. (2020).

#### Parents/carers with mental health needs

(i) Provision of/direction to self‐help materials and/or online resources
(ii) Signposting/referral to appropriate internal or external services including local Improving Access to Psychological Therapies (IAPT) services

### Outcome measures

#### Parental mental health

Parental anxiety was assessed using the 7‐item, self‐report Generalized Anxiety Disorder Scale (GAD‐7; Spitzer et al., 2006). Parental depression was assessed using the 9‐item self‐report Patient Health Questionnaire (PHQ‐9; Kroenke et al., 2001). Scores ≥10 were considered to be above the clinical threshold.

#### Child mental health and quality of life

Child mental health was assessed using the Strengths and Difficulties Questionnaire (SDQ), a parent‐report measure for children aged 4–17 (Goodman & Goodman, 2011) and child quality of life was assessed using the parent report Pediatric Quality of Life Inventory (PedsQL; Varni et al., 2001). These measures have all been shown to demonstrate good psychometric properties (Varni et al., 2001; Goodman, 2001; Plummer et al., 2016; Ueki et al., 2013). They have been validated in UK populations (Goodman & Goodman, 2011; Upton et al., 2005).

### Analysis

For all analyses, missing data at 6‐months post‐baseline were managed using multiple imputation. All subscales of the relevant scale at both time points and participant characteristics (deprivation, ethnicity and whether they received a brief psychological intervention) were included in the multiple imputation model. Ten datasets were imputed. Total scores were used for all measures. All descriptive statistics, handling of missing data and analyses were undertaken using SPSS statistical analysis software (V.25, IBM).

(1) Determining the level of mental health need in the parents attending the drop‐in centre

The percentage of parents scoring ≥10 (i.e., above clinical threshold) on measures of parental mental health was computed.

(2) Examining the association between parental mental health and child mental health and quality of life

Bivariate correlations using Spearman's rho coefficients were used to explore the associations between parent and child outcomes (de Winter et al., 2016). Correlations of .10 are considered small, .30 are considered moderate and .50 are considered large. Confidence intervals for correlations were computed using SPSS syntax.

(3) Examining the outcome of the drop‐in centre on the parents' own mental health

Descriptive statistics for GAD‐7 and PHQ‐9 total scores at baseline (time one) and 6‐months (time two) were calculated. Difference scores were based on the mean change in scores. Changes were tested using paired samples *t*‐tests and converted into standardised effect sizes (Cohen's d) where .20 is considered a small effect, .50 a medium effect, and .80 a large effect (Cohen, 2013). A sensitivity analysis was conducted in which parents who had been the primary recipient of the intervention were removed.

## RESULTS

### Participant flow

**FIGURE 1:**
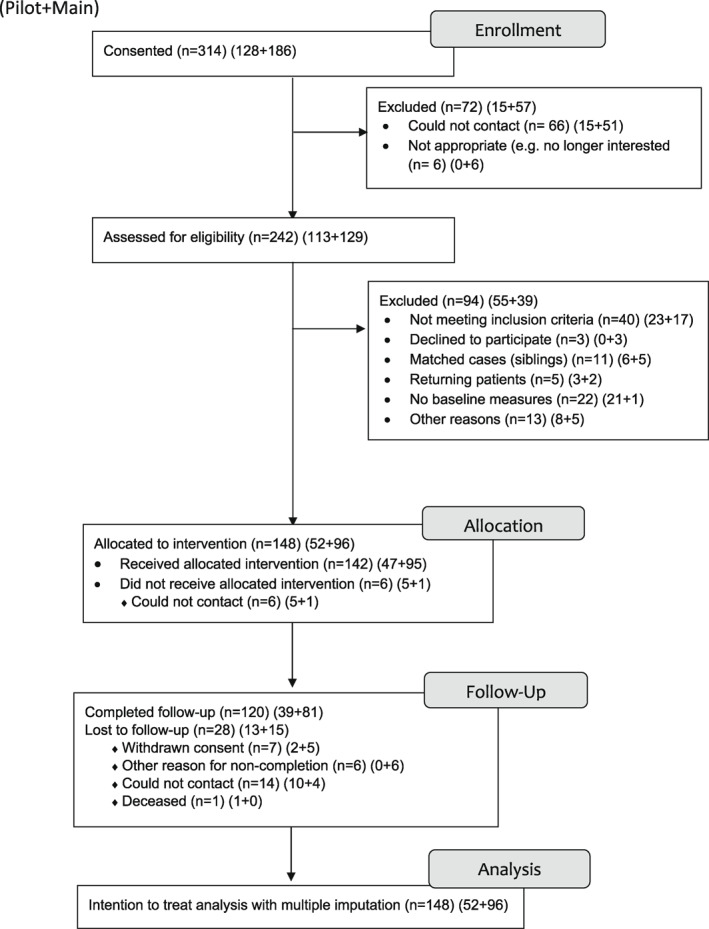
Adapted CONSORT diagram showing patient flow

Three hundred and fourteen participants initially consented to take part. No children or young people came to seek the service without a carer/parent. One hundred and eighty‐six participants were allocated to the intervention. One hundred and thirty‐nine of those attending had primary concerns relating to the hospital patient (i.e., child or young person), 36 had primary concerns relating to a parent of a hospital patient and 18 had primary concerns relating to a sibling. The demographics of the 186 participants are reported elsewhere (Catanzano et al., 2020). Figure 1 illustrates the flow of participants through the study, with reasons for exclusion/attrition at each stage of the pathway. Analyses were completed for all parents and for their child who was the primary participant (either patient or patient's sibling). Eleven families of those 186 allocated to an intervention included more than one participant. In these 11 families, only the parent and patient data were included and the sibling data were removed. Returning patients (*n* = 5) and those with no baseline measures (*n* = 22) were also removed, leaving a sample size of 148 in the analysis (Figure 1). Of the 148 parents who had baseline data for both GAD‐7 and PHQ‐9, 120 parents completed the GAD‐7 at 6‐months post‐baseline and 118 parents completed the PHQ‐9 at 6‐months post‐baseline. Twenty‐eight 6‐month post‐baseline scores were imputed for GAD‐7 and 30 for PHQ‐9.

### Participant and intervention characteristics

The full characteristics of the initial 186 participants in the main ‘drop‐in centre’ study are reported elsewhere (Catanzano et al., 2020). For the 148 families with paired parent and child data that were included in the analysis, the recipient of the intervention was a hospital patient (*n* = 112), a parent (*n* = 30) or a sibling (*n* = 14). This includes eight families where both parent and child were a recipient of the intervention (i.e., Four parent and sibling; four parent and patient). One hundred and thirty‐two of the 148 parents were mothers and 16 were fathers (mean age 40.64 years, SD = 7.99 years). Fifty nine percent were married, 63% were employed (31% full‐time; 25% part‐time; 7% self‐employed) and 12% had a self‐reported disability.

Participants were from a number of hospital departments, most commonly rheumatology (14%), ophthalmology (14%) and neurology (11%). See Table S1 for details of the main medical conditions of participants. According to the standardised measures, the 148 children and young people presented with elevated levels of: emotional problems (58%), conduct problems (39%), hyperactivity (34%) and peer problems (36%). The 148 parents presented with elevated levels of: anxiety only (13.5%), depression only (6%), both anxiety and depression (34.5%), anxiety with or without depression (48%), depression with or without anxiety (41%).

Following initial assessment, considering interventions delivered for child mental health problems, 41 of the 148 participants were provided with a brief modified version of the modular psychological treatment MATCH‐ADTC, 55 were referred onwards, 10 had liaison work, 5 underwent a neurodevelopmental assessment and 44 were signposted to resources/services. Of the five children and young people who underwent a neurodevelopmental assessment with a consultant child and adolescent psychiatrist, two were diagnosed with autism spectrum disorder, one with attention deficit hyperactivity disorder, and one with intellectual disability. The one other participant was not given a diagnosis from the neurodevelopmental assessment. Participants could be provided with more than one type of intervention. See Catanzano et al. (2020) for further details.

Thirty of the 148 parents received an intervention for their own mental health. Thirteen of these 30 were referred to internal psychological services. Twelve referrals were accepted, nine had been assessed, six had started treatment and three families had declined the service by the time of 6‐months post‐baseline. They were treated by: Family therapists (2), Clinical psychologists (3), and Assistant psychologists (1). The type of intervention included: high intensity cognitive behavioural therapy (CBT) (2), family therapy/other (2) and low intensity CBT (2). The number of treatment sessions delivered by the 6‐month post‐baseline point ranged between 4 and 24. Thirteen parents were signposted to adult Improving Access to Psychological Therapies (IAPT) services, with one refusing support and another declining both the treatments offered (computerised CBT as they did not think this would be helpful and subsequently phone CBT as this clashed with their working hours) following assessment from their local IAPT. Of the remaining 11, one has been recorded as having had three therapy sessions and three as not being seen by IAPT by the 6‐month post‐baseline time point. The outcome of the IAPT signposting is unknown for the remaining seven. The remaining four parents received single‐session counselling (1), were signposted to either self‐help materials (1), other services (1), or to a psychologist already involved with another member of the family (1).

### Outcomes

(1) Determining the level of mental health need in the parents attending the drop‐in centre

Mean scores on the GAD‐7 and PHQ‐9 are presented in Table 1. The results indicate an elevation in both parental anxiety and depression with 48% of the 148 parents scoring above clinical threshold for anxiety and 41% for depression at baseline, compared to norms of approximately 6% for anxiety (Hinz et al., 2017) and 7% for depression (Rief et al., 2004) in the general population.

(2) Examining the association between parental mental health and child mental health and quality of life

**TABLE 1:** Comparison of GAD‐7 and PHQ‐9 parent‐reported scores at baseline and 6‐months post‐baseline

Measure	*n*	Baseline	6‐months Post‐baseline	Mean difference (CI)	*P*	*d*	df^a^
		*M* (SE)	*M* (SE)				
GAD total score (all participants)	148	9.20 (0.51)	6.91 (0.48)	2.29 (1.22; 3.36)	<.001***	.38	78
PHQ total score (all participants)	148	8.78 (0.55)	6.97 (0.52)	1.81 (0.64; 3.00)	.003**	.28	85
GAD total score (parent is intervention recipient removed)	118	8.39 (0.55)	6.32 (0.56)	2.07 (0.94; 3.21)	<.001***	.02	70
PHQ total score (parent is intervention recipient removed)	118	7.76 (0.56)	6.57 (0.58)	1.19 (0.03; 2.34)	.044*	.01	73

*Note*: Multiple imputations by fully conditional specification was used to account for missing data and degrees of freedom (df) were adjusted accordingly. Means (*M*), standard errors (SE), 95% confidence intervals (CI) around the mean difference, *p* values for paired *t*‐tests and effect sizes (*d*) are shown for all participants included in the analysis.

Abbreviation: CI, 95% Confidence Interval for Mean Difference.

^a^ Barnard J, Rubin DB. Small‐Sample Degrees of Freedom with Multiple Imputation. Biometrika.1999; 86(4):948–55.

*p < .05; **p < .01; ***p < .001.

Parent‐reported child mental health at baseline, as measured by the total score on the SDQ, was moderately positively correlated with parental anxiety as measured by the total score on the GAD‐7 (*r*_s_ = .26, *p* = .002, *N* = 140; See Table 2) and parental low mood as measured by the total score on the PHQ‐9 (*r*_s_ = .29, *p* = .001, *N* = 140), at baseline, Child quality of life reported at baseline, as measured by the total score on the PedsQL, was correlated with parental anxiety (*r*_s_ = −.21, *p* = .045, *N* = 91) and low mood at baseline (*r*_s_ = −0.22, *p* = .040, *N* = 91). Correlations of .1 are considered to be small and those of .3 are moderate.

**TABLE 2:** Correlations between parental mental health and child wellbeing and quality of life scores at baseline

	1	2	3	4	5	6	7	8
1. GAD‐7 total score at baseline								
2. PHQ‐9 total score at baseline	0.816 (0.74; 0.87)							
3. SDQ total score at baseline	0.263 (0.10; 0.41)	0.288 (0.12; 0.44)						
4. PedsQL total score at baseline	−0.210 (−0.40; 0.00)	−0.215 (−0.40;‐0.01)	−0.405 (−0.57;‐0.21)					
5. Difference in GAD‐7 total scores	0.538 (0.40; 0.65)	0.367 (0.21; 0.50)	0.080 (−0.09; 0.24)	−0.116 (−0.31; 0.09)				
6. Difference in PHQ‐9 total scores	0.378 (0.23; 0.51)	0.507 (0.37; 0.62)	0.107 (−0.06; 0.27)	−0.084 (−0.28; 0.12)	0.620 (0.50; 0.72)			
7. Difference in SDQ total scores	0.139 (−0.03; 0.30)	0.053 (−0.12; 0.22)	0.355 (0.19; 0.50)	−0.137 (−0.33; 0.07)	0.115 (−0.05; 0.28)	0.098 (−0.07; 0.26)		
8. Difference in PedsQL total scores	−0.171 (−0.36; 0.04)	−0.097 (−0.30; 0.11)	−0.071 (−0.27; 0.14)	0.305 (0.10; 0.48)	−0.138 (−0.33; 0.07)	0.087 (−0.12; 0.29)	−0.162 (−0.36; 0.05)	

*Note*: Multiple imputations by fully conditional specification was used to account for missing data. Spearman's rho correlation coefficient (*r*_s_) and 95% confidence intervals (CI) are shown for all participants included in the analysis. 1 = small effect size; .3 = moderate effect size; .5 = large effect size.

Abbreviation: PedsQL, Pediatric Quality of Life Inventory; SDQ, Strengths and Difficulties Questionnaire.

There was a small correlation between change in parent reported child wellbeing (SDQ total) between baseline and 6‐months post‐baseline and changes in parental low mood and anxiety between baseline and 6‐months post‐baseline (*r*_s_ = .10, *p* = .306, *N* = 137 and *r*_s_ = .12, *p* = .242, *N* = 137, respectively). There was also a small correlation between change in parent reported child quality of life (PedsQL total) between baseline and 6‐months post‐baseline and changes in parental low mood and anxiety between baseline and 6‐month post‐baseline (*r*_s_ = .09, *p* = .454, *N* = 92 and *r*_s_ = −.14, *p* = .231, *N* = 92, respectively).

(3) Examining the outcome of the drop‐in centre on the parents' own mental health

Self‐reported parental anxiety demonstrated a decrease from an estimated mean score of 9.20 (0.51) at baseline to 6.91 (0.48) at 6‐months post‐baseline, a mean decrease of 2.29, 95% CI (1.22–3.36), *t*(78) = 4.20, *p* < .001, *d* = 0.38 (small effect size; see Table 1). Self‐reported parental low mood also demonstrated a decrease from an estimated mean score of 8.78 (0.55) at baseline to 6.97 (0.52) at 6‐months post‐baseline, a mean decrease of 1.81, 95% CI (0.64–3.00), *t*(85) = 3.02, *p* = .003, *d* = 0.28 (small effect size). When the 30 parents who were the recipients of intervention were removed from these analyses, the pattern of results remained the same (Table 1).

## DISCUSSION

This study found that parents of children with mental health problems in the context of physical health difficulties have significant mental health problems, with almost half reaching caseness on standardised measures of anxiety and depression. The high proportion of parents reaching caseness in this study is higher than has been reported in studies of children with physical health problems alone (Pinquart, 2019a, 2019b; van Oers et al., 2014) and of children with mental health problems alone (Campbell et al., 2020). The mental health of parents attending the drop‐in centre improved across time, both for all parents attending the drop‐in centre and for those parents who received a signposting intervention or referral for their own mental health. Together the findings suggest that having a child with comorbid physical and mental difficulties places a toll on parental mental health and that attendance at a drop‐in centre may be associated with improvement in parent mental health. However, whilst parent and child mental health were moderately correlated at baseline, change in parent mental health and change in child mental health demonstrated only a small correlation, therefore the nature of the relationship between attendance at the drop‐in centre and parent and child mental health is unclear in this study.

The findings demonstrate similar effect sizes to studies showing parent mental health improves as a function of child mental health (Creswell, Violato, et al., 2020; Lavallee et al., 2019; Silverman et al., 2009) and shows that the findings extend to children and young people with physical health comorbidity. The correlational findings are also consistent with studies showing a relationship between parent and child mental health in the general population (Bagner et al., 2013; Stone et al., 2016) and with research demonstrating that treating maternal depression improves childhood depression (Wickramaratne et al., 2011). Similarly, when a parent has an anxiety disorder, rates of child recovery from the anxiety disorder is approximately half compared to when there is no parental anxiety disorder (Bodden et al., 2008; Hudson et al., 2014). This study suggests this relationship between parent and child mental health extends to the paediatric chronic health population, where parents have elevated mental health problems, which may further compound the vulnerability of this population to mental health difficulties.

Overall, it is clear that it is important to assess parent mental health and to offer signposting to self‐help materials and signposting/referrals to services that can support them as part of the child's care if needed. The benefits of providing effective, low intensity interventions for children and adults are that they are cost‐effective, acceptable, increase access and facilitate early intervention with benefits for the parent and the child (Catanzano et al., 2020; Creswell, Waite, et al., 2020; Robinson et al., 2020). It is notable that child centred CBT for anxiety does involve parents at times to support with key strategies such as exposure and reinforcement and that that intervention reduces anxiety in parents (Cobham et al., 1998). However, the reduction in parental anxiety during such child centred CBT is less than when additional mental health support is provided during the therapy itself (Creswell et al., 2015). The numbers of parents receiving direct support in the present study were too small to statistically compare with those who did not, but the findings appear similar for this group of parents with children who have physical health comorbidities. It may be the case that a stepped care approach to parent support might be helpful during treatment of child mental health difficulties, as parents with significant need may benefit from more direct support. These should be integrated into the child's care to reduce barriers such as caregiving demands, stigma, and parents prioritising their child's needs above their own (Rotberg et al., 2020). Another challenge in offering interventions for parental mental health difficulties as part of routine care for children with comorbidity is that it is the child, not the parent, that is the patient. Issues such as record keeping, data protection, communication with professionals all present important ethical and governance issues that need to be addressed (Molitor & Dvorsky, 2018).

Although the findings are novel and important, there are some limitations to the current study. The sample is relatively small which limits power. The research design is not a controlled comparison, so further research is needed to identify any specific mechanisms behind change and establish the impact of child treatment for parental mental health. It may be the case that even in the group of parents who did not receive direct intervention for their own mental health, the change in parent mental health is due to parent involvement in their child's intervention rather than directly due to a change in the child's mental health. It may also be due to change in their child's physical health condition, thereby reducing strain on the family. It could also reflect regression to the mean for both parents and children. The sample size was too small to look at differences between parental mental health in children whose mental health scores improved and in children whose scores did not, or to further investigate indicators of stasis or change in physical condition over that time for example. Comparisons to usual care as well as psychological interventions targeting parental wellbeing and child‐centred treatment with and without parent involvement would help identify mechanism of change. Importantly, the child mental health outcome is parent‐report scores on the SDQ which is likely to be confounded by parental mental health and future research should aim for independent reporting of child and parent mental health. We did not have full diagnostic measures of either child or parent mental health and future research may benefit from full diagnostic interviews. Additionally, it is important to draw a distinction between statistically significant findings and those that are clinically meaningful to individual patients. Finally, the vast majority of parents were mothers and understanding the role of paternal mental health and its interaction with maternal and child mental health is critical to be able to optimise the impact of interventions for the whole family.

In conclusion, parents of children with comorbid physical and mental health needs who attended a drop‐in wellbeing centre have elevated levels of anxiety and depression. These mental health problems are associated with their child's mental health at baseline, and improved after their child attended the drop‐in mental health centre that provided a low‐intensity intervention for the child and/or parent. The uncontrolled nature of the study limits the extent to which we can attribute the change in parental mental health to specific interventions for child or parent mental health and further research is needed to understand this association. However, the results suggest that assessing and addressing the mental health of parents of children with comorbidity as part of routine treatment for the child's mental health may bring important benefits to parents and children. This should be organised on site and integrated into paediatric health care.

## CONFLICT OF INTEREST

The authors have declared that they have no competing or potential conflicts of interest.

## ETHICS STATEMENT

Ethics approval was granted by the London Riverside Research Ethics Committee (REC reference number: 16/LO/1915).

## AUTHOR CONTRIBUTIONS

Conceptualization, Sophie D. Bennett, Eleanor Kerry, Anna E. Coughtrey, Isobel Heyman and Roz Shafran; Data curation, Matteo Catanzano, Eleanor Kerry, Natalia Rojas; Formal analysis, Eleanor Kerry, Charlotte Sanderson, Kate Fifield, Brian C. F. Ching, Matteo Catanzano, Natalia Rojas, Sophie D. Bennett; Funding acquisition, Sophie D. Bennett, Anna E. Coughtrey, Isobel Heyman and Roz Shafran; Methodology, Sophie D. Bennett, Eleanor Kerry, Matteo Catanzano, Roz Shafran; Supervision, Anna E. Coughtrey, Sophie D. Bennett, Holan Liang, Roz Shafran, Isobel Heyman; Writing—original draft, Eleanor Kerry, Sophie D. Bennett, Roz Shafran, Writing—review & editing – all authors. All authors have read and agreed to the published version of the manuscript.

## Supporting information

Table S1

## Data Availability

The data that support the findings of this study are available on request from the corresponding author. The data are not publicly available due to privacy or ethical restrictions.

## References

[jcv212046-bib-0001] Acri, M. C. , & Hoagwood, K. E. (2015). Addressing parental mental health within interventions for children: A review. Research on Social Work Practice, 25(5), 578–586. 10.1177/1049731514546027 26527857 PMC4627715

[jcv212046-bib-0002] Anderson, J. K. , Howarth, E. , Vainre, M. , Jones, P. B. , & Humphrey, A. (2017). A scoping literature review of service‐level barriers for access and engagement with mental health services for children and young people. Children and Youth Services Review, 77, 164–176. 10.1016/j.childyouth.2017.04.017

[jcv212046-bib-0003] Arango, C. , Díaz‐Caneja, C. M. , McGorry, P. D. , Rapoport, J. , Sommer, I. E. , Vorstman, J. A. , McDaid, D. , Marín, O. , Serrano‐Drozdowskyj, E. , Freedman, R. , & Carpenter, W. (2018). Preventive strategies for mental health. The Lancet Psychiatry, 5(7), 591–604. 10.1016/s2215-0366(18)30057-9 29773478

[jcv212046-bib-0004] Bagner, D. M. , Pettit, J. W. , Lewinsohn, P. M. , Seeley, J. R. , & Jaccard, J. (2013). Disentangling the temporal relationship between parental depressive symptoms and early child behavior problems: A transactional framework. Journal of Clinical Child and Adolescent Psychology, 42(1), 78–90. 10.1080/15374416.2012.715368 22963145 PMC4399760

[jcv212046-bib-0005] Barlow, J. H. , & Ellard, D. R. (2006). The psychosocial well‐being of children with chronic disease, their parents and siblings: An overview of the research evidence base. Child: Care, Health and Development, 32(1), 19–31. 10.1111/j.1365-2214.2006.00591.x 16398788

[jcv212046-bib-0006] Barwick, M. , Urajnik, D. , Sumner, L. , Cohen, S. , Reid, G. , Engel, K. , & Moore, J. E. (2013). Profiles and service utilization for children accessing a mental health walk‐in clinic versus usual care. Journal of Evidence‐Based Social Work, 10(4), 338–352. 10.1080/15433714.2012.663676 23879357

[jcv212046-bib-0007] Bennett, S. , Shafran, R. , Coughtrey, A. , Walker, S. , & Heyman, I. (2015). Psychological interventions for mental health disorders in children with chronic physical illness: A systematic review. Archives of Disease in Childhood, 100(4), 308–316. 10.1136/archdischild-2014-307474 25784736

[jcv212046-bib-0008] Bennett, S. D. , Coughtrey, A. E. , Heyman, I. , Greally, S. , Clarkson, H. , Bhattacharyya, T. , Lewis, C. , Varadkar, S. , & Shafran, R. (2018). Guided self‐help for mental health disorders in children and young people with chronic neurological conditions: A qualitative evaluation. European Journal of Paediatric Neurology, 22(4), 620–631. 10.1016/j.ejpn.2018.02.011 29631920

[jcv212046-bib-0009] Bodden, D. H. , Bogels, S. M. , Nauta, M. H. , De Haan, E. , Ringrose, J. , Appelboom, C. , Brinkman, A. G. , & Appelboom‐Geerts, K. C. (2008). Child versus family cognitive‐behavioral therapy in clinically anxious youth: An efficacy and partial effectiveness study. Journal of the American Academy of Child & Adolescent Psychiatry, 47(12), 1384–1394. 10.1097/CHI.0b013e318189148e 18981932

[jcv212046-bib-0010] Campbell, T. C. H. , Reupert, A. , Sutton, K. , Basu, S. , Davidson, G. , Middeldorp, C. M. , Naughton, M. , & Maybery, D. (2020). Prevalence of mental illness among parents of children receiving treatment within child and adolescent mental health services (CAMHS): A scoping review. European Child and Adolescent Psychiatry, 30, 997–1012. 10.1007/s00787-020-01502-x 32133563

[jcv212046-bib-0011] Catanzano, M. , Bennett, S. D. , Kerry, E. , Liang, H. , Heyman, I. , Coughtrey, A. E. , Fifield, K. , Taylor, C. , Dalgleish, T. , Xu, L. , & Shafran, R. (2020). Evaluation of a mental health drop‐in centre offering brief transdiagnostic psychological assessment and treatment for children and adolescents with long‐term physical conditions and their families: A single‐arm, open, non‐randomised trial. Evidence‐Based Mental Health, 24, 25–32. 10.1136/ebmental-2020-300197 33243761 PMC7958088

[jcv212046-bib-0012] Chorpita, B. , & Weisz, J. R. (2009). Modular approach to therapy for children with anxiety, depression, trauma, or conduct problems (MATCH‐ADTC). PracticeWise, LLC.

[jcv212046-bib-0013] Cobham, V. E. , Dadds, M. R. , & Spence, S. H. (1998). The role of parental anxiety in the treatment of childhood anxiety. Journal of Consulting and Clinical Psychology, 66(6), 893–905. 10.1037//0022-006x.66.6.893 9874902

[jcv212046-bib-0014] Cohen, J. (2013). Statistical power analysis for the behavioral sciences. Academic Press.

[jcv212046-bib-0015] Cousino, M. K. , & Hazen, R. A. (2013). Parenting stress among caregivers of children with chronic illness: A systematic review. Journal of Pediatric Psychology, 38(8), 809–828. 10.1093/jpepsy/jst049 23843630

[jcv212046-bib-0016] Crenna‐Jennings, W. , & Hutchinson, J. (2020). Access to child and adolescent mental health services in 2019. Education Policy Institute. https://epi.org.uk/wp‐content/uploads/2020/01/Access‐to‐CAMHS‐in‐2019_EPI.pdf

[jcv212046-bib-0017] Creswell, C. , Cruddace, S. , Gerry, S. , Gitau, R. , McIntosh, E. , Mollison, J. , Murray, L. , Shafran, R. , Stein, A. , Violato, M. , Voysey, M. , Willetts, L. , Williams, N. , Yu, L. M. , & Cooper, P. J. (2015). Treatment of childhood anxiety disorder in the context of maternal anxiety disorder: A randomised controlled trial and economic analysis. Health Technology Assessment, 19(38), 1–184. vii‐viii. 10.3310/hta19380 PMC4781330 26004142

[jcv212046-bib-0018] Creswell, C. , Violato, M. , Cruddace, S. , Gerry, S. , Murray, L. , Shafran, R. , Stein, A. , Willetts, L. , McIntosh, E. , & Cooper, P. J. (2020). A randomised controlled trial of treatments of childhood anxiety disorder in the context of maternal anxiety disorder: Clinical and cost‐effectiveness outcomes. The Journal of Child Psychology and Psychiatry, 61(1), 62–76. 10.1111/jcpp.13089 31364169 PMC6916180

[jcv212046-bib-0019] Creswell, C. , Waite, P. , & Hudson, J. (2020). Practitioner Review: Anxiety disorders in children and young people—Assessment and treatment. The Journal of Child Psychology and Psychiatry, 61(6), 628–643. 10.1111/jcpp.13186 31960440

[jcv212046-bib-0020] de Winter, J. C. , Gosling, S. D. , & Potter, J. (2016). Comparing the Pearson and Spearman correlation coefficients across distributions and sample sizes: A tutorial using simulations and empirical data. Psychological Methods, 21(3), 273–290. 10.1037/met0000079 27213982

[jcv212046-bib-0021] D'Onofrio, B. M. , Slutske, W. S. , Turkheimer, E. , Emery, R. E. , Harden, K. P. , Heath, A. C. , Madden, P. A. , & Martin, N. G. (2007). Intergenerational transmission of childhood conduct problems: A Children of Twins Study. Archives of General Psychiatry, 64(7), 820–829. 10.1001/archpsyc.64.7.820 17606816 PMC2965630

[jcv212046-bib-0022] Eley, T. C. , McAdams, T. A. , Rijsdijk, F. V. , Lichtenstein, P. , Narusyte, J. , Reiss, D. , Spotts, E. L. , Ganiban, J. M. , & Neiderhiser, J. M. (2015). The intergenerational transmission of anxiety: A children‐of‐twins study. The American Journal of Psychiatry, 172(7), 630–637. 10.1176/appi.ajp.2015.14070818 25906669 PMC8515953

[jcv212046-bib-0023] Goodman, A. , & Goodman, R. (2011). Population mean scores predict child mental disorder rates: Validating SDQ prevalence estimators in Britain. The Journal of Child Psychology and Psychiatry, 52(1), 100–108. 10.1111/j.1469-7610.2010.02278.x 20722879

[jcv212046-bib-0024] Goodman, R. (2001). Psychometric properties of the strengths and difficulties questionnaire. Journal of the American Academy of Child & Adolescent Psychiatry, 40(11), 1337–1345. 10.1097/00004583-200111000-00015 11699809

[jcv212046-bib-0025] Goodman, R. , Ford, T. , Richards, H. , Gatward, R. , & Meltzer, H. (2000). The development and well‐being assessment: Description and initial validation of an integrated assessment of child and adolescent psychopathology. Journal of Child Psychology and Psychiatry, 41(5), 645–655. 10.1111/j.1469-7610.2000.tb02345.x 10946756

[jcv212046-bib-0026] Hinz, A. , Klein, A. M. , Brahler, E. , Glaesmer, H. , Luck, T. , Riedel‐Heller, S. G. , Wirkner, K. , & Hilbert, A. (2017). Psychometric evaluation of the Generalized Anxiety Disorder Screener GAD‐7, based on a large German general population sample. Journal of Affective Disorders, 210, 338–344. 10.1016/j.jad.2016.12.012 28088111

[jcv212046-bib-0027] Holmbeck, G. N. , Johnson, S. Z. , Wills, K. E. , McKernon, W. , Rose, B. , Erklin, S. , & Kemper, T. (2002). Observed and perceived parental overprotection in relation to psychosocial adjustment in preadolescents with a physical disability: The mediational role of behavioral autonomy. Journal of Consulting and Clinical Psychology, 70(1), 96–110. 10.1037//0022-006x.70.1.96 11860060

[jcv212046-bib-0028] Hood, K. K. , Huestis, S. , Maher, A. , Butler, D. , Volkening, L. , & Laffel, L. M. (2006). Depressive symptoms in children and adolescents with type 1 diabetes: Association with diabetes‐specific characteristics. Diabetes Care, 29(6), 1389–1389. 10.2337/dc06-0087 16732028

[jcv212046-bib-0029] Hudson, J. L. , Newall, C. , Rapee, R. M. , Lyneham, H. J. , Schniering, C. C. , Wuthrich, V. M. , Schneider, S. , Seeley‐Wait, E. , Edwards, S. , & Gar, N. S. (2014). The impact of brief parental anxiety management on child anxiety treatment outcomes: A controlled trial. Journal of Clinical Child and Adolescent Psychology, 43(3), 370–380. 10.1080/15374416.2013.807734 23845064 PMC4037847

[jcv212046-bib-0030] Hysing, M. , Elgen, I. , Gillberg, C. , Lie, S. A. , & Lundervold, A. J. (2007). Chronic physical illness and mental health in children. Results from a large‐scale population study. The Journal of Child Psychology and Psychiatry, 48(8), 785–792. 10.1111/j.1469-7610.2007.01755.x 17683450

[jcv212046-bib-0031] Jarosz, E. , & Bayer, J. K. (2017). Service evaluation of the Cool Little Kids parenting program delivered in the community. Advances in Mental Health, 17(1), 6–20. 10.1080/18387357.2017.1418630

[jcv212046-bib-0032] Knapp, M. , McDaid, D. , & Parsonage, M. (2011). Mental health promotion and mental illness prevention: The economic case. The Department of Health. https://assets.publishing.service.gov.uk/government/uploads/system/uploads/attachment_data/file/215626/dh_126386.pdf

[jcv212046-bib-0033] Kroenke, K. , Spitzer, R. L. , & Williams, J. B. (2001). The PHQ‐9: Validity of a brief depression severity measure. Journal of General Internal Medicine, 16(9), 606–613. 10.1046/j.1525-1497.2001.016009606.x 11556941 PMC1495268

[jcv212046-bib-0034] Lavallee, K. , Schuck, K. , Blatter‐Meunier, J. , & Schneider, S. (2019). Transgenerational improvements following child anxiety treatment: An exploratory examination. PLoS One, 14(2), e0212667. 10.1371/journal.pone.0212667 30817752 PMC6394948

[jcv212046-bib-0035] Law, E. , Fisher, E. , Eccleston, C. , & Palermo, T. M. (2019). Psychological interventions for parents of children and adolescents with chronic illness. Cochrane Database of Systematic Reviews, 3, CD009660. 10.1002/14651858.CD009660.pub4 30883665 PMC6450193

[jcv212046-bib-0036] Lima, N. N. , do Nascimento, V. B. , de Carvalho, S. M. , de Abreu, L. C. , Neto, M. L. , Brasil, A. Q. , Junior, F. T. , de Oliveira, G. F. , & Reis, A. O. (2013). Childhood depression: A systematic review. Neuropsychiatric Disease and Treatment, 9, 1417–1425. 10.2147/NDT.S42402 24092979 PMC3788699

[jcv212046-bib-0037] McAdams, T. A. , Rijsdijk, F. V. , Neiderhiser, J. M. , Narusyte, J. , Shaw, D. S. , Natsuaki, M. N. , Spotts, E. L. , Ganiban, J. M. , Reiss, D. , Leve, L. D. , Lichtenstein, P. , & Eley, T. C. (2015). The relationship between parental depressive symptoms and offspring psychopathology: Evidence from a children‐of‐twins study and an adoption study. Psychological Medicine, 45(12), 2583–2594. 10.1017/S0033291715000501 25994116 PMC4523449

[jcv212046-bib-0038] Miller, A. H. , Ancoli‐Israel, S. , Bower, J. E. , Capuron, L. , & Irwin, M. R. (2008). Neuroendocrine‐immune mechanisms of behavioral comorbidities in patients with cancer. Journal of Clinical Oncology, 26(6), 971–982. 10.1200/JCO.2007.10.7805 18281672 PMC2770012

[jcv212046-bib-0039] Molitor, S. J. , & Dvorsky, M. R. (2018). Ethical considerations for assessing parent mental health during child assessment services. Ethics & Behavior, 29(2), 87–100. 10.1080/10508422.2018.1482746 34168418 PMC8220894

[jcv212046-bib-0040] Nath, R. , Shannon, H. , Georgiades, K. , Sword, W. , & Raina, P. (2016). The impact of drop‐in centers on the health of street children in New Delhi, India: A cross‐sectional study. Child Abuse & Neglect, 62, 122–131. 10.1016/j.chiabu.2016.11.001 27837694

[jcv212046-bib-0041] Naylor, C. , Parsonage, M. , McDaid, D. , Knapp, M. , Fossey, M. , & Galea, A. (2012). Long‐term conditions and mental health: The cost of co‐morbidities. The King’s Fund and Centre for Mental Health. https://www.kingsfund.org.uk/sites/default/files/field/field_publication_file/long‐term‐conditions‐mental‐health‐cost‐comorbidities‐naylor‐feb12.pdf

[jcv212046-bib-0042] Neece, C. L. (2014). Mindfulness‐based stress reduction for parents of young children with developmental delays: Implications for parental mental health and child behavior problems. Journal of Applied Research in Intellectual Disabilities, 27(2), 174–186. 10.1111/jar.12064 23813562

[jcv212046-bib-0043] Pierce, M. , Hope, H. F. , Kolade, A. , Gellatly, J. , Osam, C. S. , Perchard, R. , Kosidou, K. , Dalman, C. , Morgan, V. , Di Prinzio, P. , & Abel, K. M. (2020). Effects of parental mental illness on children's physical health: Systematic review and meta‐analysis. British Journal of Psychiatry, 217(1), 354–363. 10.1192/bjp.2019.216 31610824

[jcv212046-bib-0044] Pinquart, M. (2019a). Featured article: Depressive symptoms in parents of children with chronic health conditions: A meta‐analysis. Journal of Pediatric Psychology, 44(2), 139–149. 10.1093/jpepsy/jsy075 30346613

[jcv212046-bib-0045] Pinquart, M. (2019b). Meta‐analysis of anxiety in parents of young people with chronic health conditions. Journal of Pediatric Psychology, 44(8), 959–969. 10.1093/jpepsy/jsz024 31220871

[jcv212046-bib-0046] Pinquart, M. , & Shen, Y. (2011a). Behavior problems in children and adolescents with chronic physical illness: A meta‐analysis. Journal of Pediatric Psychology, 36(9), 1003–1016. 10.1093/jpepsy/jsr042 21810623

[jcv212046-bib-0047] Pinquart, M. , & Shen, Y. (2011b). Depressive symptoms in children and adolescents with chronic physical illness: An updated meta‐analysis. Journal of Pediatric Psychology, 36(4), 375–384. 10.1093/jpepsy/jsq104 21088072

[jcv212046-bib-0048] Pinquart, M. , & Shen, Y. (2011c). Anxiety in children and adolescents with chronic physical illnesses: A meta‐analysis. Acta Paediatrica, 100(8), 1069–1076. 10.1111/j.1651-2227.2011.02223.x 21332786

[jcv212046-bib-0049] Plummer, F. , Manea, L. , Trepel, D. , & McMillan, D. (2016). Screening for anxiety disorders with the GAD‐7 and GAD‐2: A systematic review and diagnostic metaanalysis. General Hospital Psychiatry, 39, 24–31. 10.1016/j.genhosppsych.2015.11.005 26719105

[jcv212046-bib-0050] Reupert, A. E. , Maybery, D. J. , & J Maybery, N. M. (2013). Children whose parents have a mental illness: Prevalence, need and treatment. Medical Journal of Australia, 199(3 Suppl), S7–S9. 10.5694/mja11.11200 25369850

[jcv212046-bib-0051] Rief, W. , Nanke, A. , Klaiberg, A. , & Braehler, E. (2004). Base rates for panic and depression according to the Brief Patient Health Questionnaire: A population‐based study. Journal of Affective Disorders, 82(2), 271–276. 10.1016/j.jad.2003.11.006 15488257

[jcv212046-bib-0052] Robinson, L. , Delgadillo, J. , & Kellett, S. (2020). The dose‐response effect in routinely delivered psychological therapies: A systematic review. Psychotherapy Research, 30(1), 79–96. 10.1080/10503307.2019.1566676 30661486

[jcv212046-bib-0053] Rotberg, B. , Wittenberg, J. , Orkin, J. , Saunders, N. R. , & Cohen, E. (2020). Caring about caregivers: The role of paediatricians in supporting the mental health of parents of children with high caregiving needs. Archives of Disease in Childhood, 105(11), 1028–1030. 10.1136/archdischild-2019-318729 32376696

[jcv212046-bib-0054] Sandstrom, M. J. , & Schanberg, L. E. (2004). Peer rejection, social behavior, and psychological adjustment in children with juvenile rheumatic disease. Journal of Pediatric Psychology, 29(1), 29–34. 10.1093/jpepsy/jsh004 14747363

[jcv212046-bib-0055] Sav, A. , King, M. A. , Whitty, J. A. , Kendall, E. , McMillan, S. S. , Kelly, F. , Hunter, B. , & Wheeler, A. J. (2015). Burden of treatment for chronic illness: A concept analysis and review of the literature. Health Expectations, 18(3), 312–324. 10.1111/hex.12046 23363080 PMC5060781

[jcv212046-bib-0056] Settipani, C. A. , Hawke, L. D. , Cleverley, K. , Chaim, G. , Cheung, A. , Mehra, K. , Rice, M. , Szatmari, P. , & Henderson, J. (2019). Key attributes of integrated community‐based youth service hubs for mental health: A scoping review. International Journal of Mental Health Systems, 13, 52. 10.1186/s13033-019-0306-7 31367230 PMC6651922

[jcv212046-bib-0057] Silverman, W. K. , Kurtines, W. M. , Jaccard, J. , & Pina, A. A. (2009). Directionality of change in youth anxiety treatment involving parents: An initial examination. Journal of Consulting and Clinical Psychology, 77(3), 474–485. 10.1037/a0015761 19485589 PMC2778202

[jcv212046-bib-0058] Spitzer, R. L. , Kroenke, K. , Williams, J. B. , & Lowe, B. (2006). A brief measure for assessing generalized anxiety disorder: The GAD‐7. Archives of Internal Medicine, 166(10), 1092–1097. 10.1001/archinte.166.10.1092 16717171

[jcv212046-bib-0059] Stone, L. L. , Mares, S. H. , Otten, R. , Engels, R. C. , & Janssens, J. M. (2016). The co‐development of parenting stress and childhood internalizing and externalizing problems. Journal of Psychopathology and Behavioral Assessment, 38, 76–86. 10.1007/s10862-015-9500-3 27069304 PMC4789299

[jcv212046-bib-0060] Tyler, E. T. , Hulkower, R. L. , & Kaminski, J. W. (2017). Behavioral health integration in pediatric primary care: Considerations and opportunities for policymakers, planners, and providers. Milbank Memorial Fund. https://www.milbank.org/wp‐content/uploads/2017/03/MMF_BHI_REPORT_FINAL.pdf

[jcv212046-bib-0061] Ueki, H. , Kaneko, M. , & Narukawa, M. (2013). Points to consider in multiregional trials using PHQ‐9: A meta‐analysis on PHQ‐9. Value in Health, 16(7), A541. 10.1016/j.jval.2013.08.1372

[jcv212046-bib-0062] Upton, P. , Eiser, C. , Cheung, I. , Hutchings, H. A. , Jenney, M. , Maddocks, A. , Russell, I. T. , & Williams, J. G. (2005). Measurement properties of the UK‐English version of the Pediatric Quality of Life Inventory 4.0 (PedsQL) generic core scales. Health Qual Life Outcomes, 3, 22. 10.1186/1477-7525-3-22 15804349 PMC1079918

[jcv212046-bib-0063] van Oers, H. A. , Haverman, L. , Limperg, P. F. , van Dijk‐Lokkart, E. M. , Maurice‐Stam, H. , & Grootenhuis, M. A. (2014). Anxiety and depression in mothers and fathers of a chronically ill child. Maternal and Child Health Journal, 18(8), 1993–2002. 10.1007/s10995-014-1445-8 24791971

[jcv212046-bib-0064] Varni, J. W. , Seid, M. , & Kurtin, P. S. (2001). PedsQL 4.0: Reliability and validity of the Pediatric Quality of Life Inventory version 4.0 generic core scales in healthy and patient populations. Medical Care, 39(8), 800–812. 10.1097/00005650-200108000-00006 11468499

[jcv212046-bib-0065] Wickramaratne, P. , Gameroff, M. J. , Pilowsky, D. J. , Hughes, C. W. , Garber, J. , Malloy, E. , King, C. , Cerda, G. , Sood, A. B. , Alpert, J. E. , Trivedi, M. H. , Fava, M. , Rush, A. J. , Wisniewski, S. , & Weissman, M. M. (2011). Children of depressed mothers 1 year after remission of maternal depression: Findings from the STAR*D‐Child study. The American Journal of Psychiatry, 168(6), 593–602. 10.1176/appi.ajp.2010.10010032 21406462 PMC3423977

[jcv212046-bib-0066] Wilkinson, P. O. , Harris, C. , Kelvin, R. , Dubicka, B. , & Goodyer, I. M. (2013). Associations between adolescent depression and parental mental health, before and after treatment of adolescent depression. European Child and Adolescent Psychiatry, 22(1), 3–11. 10.1007/s00787-012-0310-9 22836732

