# Supplementary material for: A drop‐in centre for treating mental health problems in children with chronic illness: Outcomes for parents and their relationship with child outcomes
Source: JCPP Adv. 2021 Oct 25;1(4):e12046. doi: 10.1002/jcv2.12046 (PMC10242943; doi:10.1002/jcv2.12046)
Supplement: Supplementary file 1 — Table S1 [file JCV2-1-e12046-s001.docx]

**Table S1: Number of participants across recorded medical conditions.** Categories were used instead of individual conditions/diseases to avoid possible identification of patients, especially with rarer conditions/diseases.

| **Main medical condition category** | **Number of participants (%)** |
| --- | --- |
| Autoimmune | 19 (12.8) |
| Genetics | 37 (25.0) |
| Medically unexplained symptoms | 14 (9.5) |
| Neurological | 21 (14.2) |
| Other | 57 (38.5) |
